# Implementing continuity of nutrition care from hospital to home for older medical inpatients with malnutrition or nutritional risk: nursing contributions, barriers, and practice gaps

**DOI:** 10.3389/fnut.2026.1980311

**Published:** 2026-10-22

**Authors:** Xieyi Wang, Hezhuo Wang

**Affiliations:** 1 Ningbo No. 6 Hospital, Ningbo, China; 2 The Affiliated People's Hospital of Ningbo University, Ningbo, China

**Keywords:** continuity of care, discharge planning, implementation science, malnutrition, nursing, nutritional care, older adults, transitional care

## Abstract

Nutrition care for older adults with malnutrition or nutritional risk frequently becomes fragmented after hospital discharge. This Mini Review examines documented continuity failures, evaluated interventions, and nursing contributions within interdisciplinary care. A focused, non-exhaustive PubMed and Europe PMC search was updated through 14 September 2026, with targeted citation checks. Evidence was selected for relevance to acute hospital-to-home care; mixed populations and adjacent settings were distinguished. No formal risk-of-bias assessment or meta-analysis was performed. Audits and qualitative studies identified gaps in discharge information, referral uptake, and support for implementing plans at home. Trials of individualized plans, home or telephone follow-up, food provision, and oral nutritional supplements showed inconsistent nutritional, functional, and service outcomes. Nursing participation was documented in some multicomponent interventions, but its independent contribution was not isolated. Attrition, unequal contact intensity, comparator care, and limited reporting of implementation outcomes constrain interpretation. Drawing on these findings, we propose a literature-informed six-step conceptual framework covering readiness and goals, portable handover, acknowledged responsibility, follow-up, escalation, and audit with feedback. Its operational measures and interdisciplinary responsibilities are specified for prospective testing, not presented as a validated pathway. Evaluation should distinguish clinical effectiveness from delivery, patient adherence, and equitable access, while incorporating patient preferences and caregiver capacity.

## 1 Introduction

Hospital discharge is a high-risk transition for older adults with malnutrition or nutritional risk. Here, malnutrition denotes an assessed condition, whereas nutritional risk denotes a screening result indicating need for further assessment; they are not interchangeable. Nutrition care encompasses assessment, planning, treatment, coordination, and monitoring. Fatigue, functional limitations, food-access problems, and uncertainty about responsibility can prevent inpatient treatment from continuing at home.

Routine-care evidence illustrates this gap. In an Australian prospective cohort of 42 older medical patients at nutritional risk, six (14%) received post-discharge dietetic review and eight (19%) practical nutrition support at home (1). A US chart review of 76 malnourished adults, not exclusively older people, documented inadequate discharge advice (2). An Australian medical/surgical audit covered 345 admissions involving 306 malnourished older patients: 35 admissions (10%) had nutrition recommendations in electronic discharge summaries and 79 (23%) had recorded dietetic follow-up within 6 months (3). These are local, documentation-dependent observations, not population prevalence estimates.

Existing reviews describe heterogeneous effects of nutritional interventions (4–6), but the links between information transfer, professional responsibilities, and sustained home care remain insufficiently synthesized. We ask: (1) which continuity failures are documented; (2) which transition components have been evaluated, with what benefits and limitations; and (3) which nursing contributions and implementation conditions are supported within interdisciplinary care? Sections 3.1–3.2 address failures, Section 3.3 compares interventions, and Sections 4–5 interpret roles and implementation conditions.

## 2 Scope and approach

This is a focused narrative Mini Review, not a systematic review. The target is older medical inpatients with malnutrition or nutritional risk discharged to a private home, including those receiving community services. Institutional rehabilitation and residential care are not the target destination. Mixed medical/surgical geriatric cohorts, broader age thresholds, and rehabilitation-to-home studies are identified as indirect evidence; their outcomes are not attributed specifically to older medical patients.

The search was updated through 14 September 2026. Four PubMed branches covered nutrition support, transitions, older age, and malnutrition, with additional nursing and dietetic terminology. All 950 returned records were exported and deduplicated to 776 PubMed records; Europe PMC returned seven records. All PubMed titles were inspected, with targeted abstract and accessible-full-text review rather than exhaustive eligibility screening. Reference lists and evidence tables of relevant reviews were checked for missed studies and companion reports. Selection prioritized relevance to the three questions, intervention contrast, documentation of delivery, and informative negative findings. Supplementary material 1 provides exact strings, fields, dates, counts, selection boundaries, and source-verification scope. The 41 cited publications include contextual sources, companion reports, and a published corrigendum; they do not represent 41 independent studies.

PubMed supplied biomedical coverage and Europe PMC supported complementary retrieval and open full texts; their overlap does not establish comprehensive coverage. CINAHL, Embase, Chinese databases, and gray literature were not searched. Selection and extraction were AI-assisted and not independently duplicated. No formal risk-of-bias grading was undertaken; critical interpretation considered design, population, comparator, attrition, follow-up, effect precision, and reporting limitations. Abstract-only information was not treated as full-text verification.

We distinguish empirical findings, our interpretation, and proposed service functions. The 2022 updated Consolidated Framework for Implementation Research (CFIR) was selected to consider determinants across organizational boundaries, not to prescribe intervention steps (7). Selected constructs inform the examples in Section 5.1; no formal coding, construct frequency counts, or comparative scoring was undertaken. Implementation outcomes are distinguished from service processes and clinical outcomes (8).

## 3 The continuity chain from identification to follow-up

### 3.1 Translating inpatient assessment into a home nutrition plan

Empirical findings indicate deficiencies in portable information. In the US audit, only five of 76 malnourished adults (6.6%) had documented instructions to consume oral nutritional supplements (ONS); its mixed-age sample limits directness (2). The Australian audit also found inconsistent placement of nutrition recommendations (3). Missing documentation does not prove that no advice was given, but can obstruct transfer to other providers.

Our interpretation is that a handover needs actionable information, not simply a diagnosis. Consensus pathways support communication of assessment, treatment, and follow-up (9), while interviews with 15 older patients and nine caregivers in Norway highlighted individualized advice and involvement in food-related decisions (10). We propose transferring risk or diagnosis, agreed goals, the authorized nutrition plan, food-access barriers, referral status, and a contact for unresolved needs. Readiness checks should address affordability, shopping and cooking capacity, safe eating, health literacy, and available help. These proposed checks have not been validated as a bundle.

### 3.2 From referral to receiving care

Published pathways recommend individualized planning, dietetic referral, education, and multidisciplinary follow-up (9, 11). These are recommendations, not demonstrations of effectiveness. The Australian audit separates offered, accepted, and attended care, showing why referral placement alone is an incomplete measure (3).

Capacity, eligibility, cost, transport, digital exclusion, and patient preferences may interrupt service uptake. A 2026 hybrid implementation-effectiveness protocol proposes hospital-to-community data sharing with dietitians and meal organizations; it provides design and measurement examples, not outcome evidence (12).

We therefore propose recording the receiving service, its acknowledgment, the planned first contact, and action after rejection or failed contact. This operational definition of service ownership is our synthesis of documented coordination gaps and pathway recommendations, not a tested causal component. Declining an unsuitable plan should prompt shared review, not automatically be classified as patient non-adherence.

### 3.3 Supporting nutrition recovery after discharge

In one three-arm Danish trial reported in two papers, 208 adults aged ≥75 years living alone received a discharge diet plan, followed by dietitian home visits, telephone consultations, or no study follow-up (13, 14). Home visits reduced 90-day readmission vs. control (hazard ratio 0.4; 95% CI 0.2–0.8), whereas the telephone estimate was inconclusive (0.7; 0.4–1.3). Preservation of activities of daily living (ADL) favored home visits, but median ADL change did not differ. Interpretation is constrained by 157 completing 8-week assessment, home visits lasting 45 vs. 15 min for calls, and 88/208 participants having hip fracture. This mixed cohort cannot establish a mode effect in medical patients alone.

NOURISH (*n* = 652) evaluated specialized ONS, not closed-loop coordination: the primary death/readmission composite was not improved, despite lower 90-day mortality (4.8% vs. 9.7%) (15). Feldblum's ward-allocated trial (*n* = 259) favored individualized dietetic care but combined two control groups and had 25.8% dropout; monthly contact occurred in all groups (16). Sharma's trial (*n* = 148) found shorter index stay, without clear post-discharge clinical benefits (17). Uncontrolled or pre–post home-care programs cannot separate treatment effects from recovery or selection (18, 19).

Multicomponent care also produced mixed outcomes. Munk's trial (*n* = 191) combined discharge planning, initial food provision, electronic municipal communication, and dietitian follow-up: nutritional, functional, and quality-of-life outcomes improved, but 6-month readmission did not (risk ratio 0.96; 95% CI 0.71–1.31) (20). HOMEFOOD (*n* = 106; three companion publications) combined 6 months of counseling with free food and ONS; nutritional and functional benefits and selected service outcomes were reported (21–23). The published corrigendum to the service-outcome report found no significant differences in readmissions or length of stay at 6, 12, or 18 months; the 1-month differences depended on the definition of hospital stay (41). Free provision, small sample size, and multiple outcomes limit attribution and transferability.

Additional trials temper a uniformly favorable interpretation. Dietitian home visits improved nutritional outcomes in Beck's trials (*n* = 152 and *n* = 71), but readmission or hospitalization estimates were imprecise (24, 25). McMurdo's supplement trial (*n* = 253) improved grip strength, not disability; reported adherence was 38.2% vs. 50.0% with the lower-energy control supplement (26). Söderström's four-arm trial (*n* = 671) found no survival advantage from advice, ONS, or both despite repeated contact (27). Improved intake, weight, or strength therefore cannot be equated with reduced disability, readmission, or mortality.

Routine delivery adds a separate concern. A French retrospective home-visit program enrolled 351 older adults; 197 (56%) completed at least two visits and 149 (42%) all planned visits. More caregivers were associated with completion (28). These are completion measures among enrollees, not reach among all eligible discharges; uncontrolled improvements cannot establish effectiveness.

Across studies, contact intensity, comparator services, free products, caregiver involvement, and illness burden vary together. Nutrition plans and electronic communication were usually bundled, so their independent effects remain uncertain. Table 1 compares 13 selected randomized trials; Supplementary material 2 separates observational evidence, professional-role evidence, and proposed implementation functions.

**Table 1:** Comparative appraisal of 13 selected randomized trials informing hospital-to-home nutrition care.

Trial and setting	Intervention and comparator follow-up	Reported findings and delivery	Critical interpretation
Pedersen et al. (13, 14) Denmark; *n* = 208; ≥75; living alone; mixed geriatric cohort (42% hip fracture)	Dietitian visits or calls at weeks 1, 2, 4 vs. no study follow-up. All groups: nurse-specialist diet plan. ADL 8 weeks; readmission 90 days.	Home visits: 90-day readmission HR 0.4 (95% CI 0.2–0.8). ADL preservation favored visits; median ADL change did not. 157 completed assessment.	45-min visits vs. 15-min calls confound mode and intensity. Selected, cognitively intact population; mixed medical/surgical directness. Nursing effect not isolated.
Beck et al. (24) Denmark; *n* = 152; ≥65; geriatric medical patients at nutritional risk	Three dietitian contacts plus GP follow-up vs. GP follow-up alone. Intervention 12 weeks; readmission/mortality 26 weeks.	Weight/intake and mobility improved; readmission OR 1.62 (95% CI 0.85–3.10). Dietetic contact nearly complete; only 10% had three GP contacts.	Nutrition benefit does not establish readmission benefit. Unequal implementation of comparator co-care complicates interpretation; CI includes no effect.
Beck et al. (25) Denmark; *n* = 71; ≥70; geriatric liaison cohort including medical/surgical recruitment	Dietitian added to liaison team vs. liaison team alone. Three visits over 12 weeks; hospitalization/mortality 6 months.	Nutritional status improved. Hospitalization OR 0.367 (95% CI 0.129–1.042). 63 completed 12-week assessment; dietitian visits nearly complete.	Small sample and imprecision; cannot claim reduced hospitalization. Tests dietitian addition, not independent nursing efficacy.
Terp et al. (29) Denmark; *n* = 144; ≥65; geriatric patients at nutritional risk	Individual diet plan and visits at weeks 1, 4, 8 vs. usual care. Nutrition/function 3 months; readmission/mortality 90/120 days.	Weight +0.9 vs. −0.8 kg (*p* = 0.032); self-rated health improved. No significant function, readmission, or mortality difference. Nursing/assistant delivery described in Ingstad et al. (4).	Multicomponent intervention; provider contribution not isolated. Primary abstract verified; personnel details corroborated through scoping-review extraction, not primary full text.
McMurdo et al. (26) Scotland; *n* = 253; mean age 82; undernourished older discharges	ONS 600 kcal/day vs. 200-kcal control supplement; assessments at 8 and 16 weeks.	Barthel difference 0.28 (95% CI −0.28 to 0.84); grip +1.52 kg (0.50–2.55). Adherence 38.2% vs. 50.0%. No falls/QoL difference.	Functional domains disagree. Active comparator and low adherence matter; on-treatment weight benefit does not prove effectiveness for all patients.
Söderström et al. (27) Sweden; *n* = 671; ≥65; malnutrition/risk; home discharge	Advice, ONS, or both vs. routine care; dietitian calls across arms. Treatment 6 months; median survival follow-up 4.1 years.	No survival difference (overall log-rank *p* = 0.762). Registry outcome follow-up nearly complete. No body-weight trajectory measured in this report.	Strong survival ascertainment, but below planned recruitment and selected home-living population. Neutral survival is not evidence of no nutritional benefit.
NOURISH (15) US; *n* = 652; ≥65; malnourished patients with selected medical diagnoses	High-protein HMB-ONS vs. placebo supplement plus standard care; 90 days after discharge.	Primary death/readmission composite 26.8% vs. 31.1%, not significant. Mortality 4.8% vs. 9.7%; RR 0.49 (95% CI 0.27–0.90). Readmission and ADL not improved.	Product-specific, selected population; secondary mortality result cannot establish success of a coordination pathway or nursing role.
Feldblum et al. (16) Israel; *n* = 259; ≥65; internal medicine patients at nutritional risk	Inpatient dietetic treatment and three home visits vs. combined inpatient-only/standard-care controls. All groups contacted monthly; 6 months.	MNA change +3.01 vs. +1.81; mortality 3.8% vs. 11.6%. Overall dropout 25.8%.	Allocation by ward, merged controls, attrition, and bundled care limit causal specificity and transferability; monthly contact was not unique to intervention.
Sharma et al. (17) Australia; *n* = 148; mean age 81.8; acute medical malnutrition	Individual plan and monthly telehealth vs. nutrition care on clinician referral. Intervention 3 months; readmission to 6 months.	Shorter index stay; no clear between-group nutritional-status, QoL, mortality, or readmission benefit.	Inpatient and post-discharge components overlap. Index-stay benefit cannot establish the effect of later telephone contact.
Munk et al. (20) Denmark; *n* = 191; older patients discharged home	Plan, initial food package, municipal electronic communication, calls, and home assessment vs. standard care. 16 weeks; readmission 6 months.	Nutrition, physical function, and QoL improved; readmission RR 0.96 (95% CI 0.71–1.31). At 16 weeks 82% vs. 61% met ≥75% of energy/protein requirements.	Bundled components prevent isolation of electronic handover or contact mode. Clinical improvement differs by endpoint; process effectiveness not demonstrated separately.
HOMEFOOD (21–23, 41) Iceland; *n* = 106; >65; geriatric discharge; one trial/three papers	Five visits, three calls, free food/ONS vs. standard care. Treatment 6 months; secondary service follow-up to 18 months.	Weight, function, and QoL improved. Corrected analyses showed fewer readmissions and shorter length of stay at 1 month under some hospital-care definitions, but no significant differences at 6, 12, or 18 months. Including day-hospital post-acute rehabilitation also removed the 1-month differences. Mortality was not different.	Small sample, multiple endpoints, and resource-intensive free provision. Corrected service-outcome findings depend on the hospital-care definition and do not establish a sustained readmission benefit. Do not count companion papers as independent replications or generalize across service endpoints.
OptiNAM (36) Denmark; *n* = 130; ≥65; acutely admitted medical patients, MNA-SF ≤ 11	Dietary counseling plus tailored risk-factor interventions vs. standard care. Outcomes 8 and 16 weeks.	No QoL, weight or strength advantage. Early intake benefit not maintained at 16 weeks. Component compliance 0%−61%; loss to follow-up 29% vs. 19%.	Implementation limitations are plausible, not proven causes of null effects. Attrition and heterogeneous component exposure reduce interpretability.
van Dronkelaar et al. (37) Netherlands; *n* = 76; ≥55, median 73; hospitalized patients at nutritional risk	Intensified trans-sectorial dietetic care vs. regular care; 3 months after discharge.	No between-group SPPB, protein intake, or other outcome advantage. Overall dropout 30%; low intervention adherence reported.	Small sample and age range limit precision/directness. Improvement in both arms is not evidence that intensified care is effective.

This purposive comparison is not a systematic inventory or a formal risk-of-bias assessment. Mixed medical/surgical cohorts and broader age thresholds are identified; findings cannot automatically be attributed to the target medical population. Pedersen's two papers and HOMEFOOD's three papers each represent one trial. Reported findings/delivery are separated from our critical interpretation. Further observational evidence and source-access details are in Supplementary material 2. ADL, activities of daily living; CI, confidence interval; GP, general practitioner; HMB, β-hydroxy-β-methylbutyrate; HR, hazard ratio; MNA, Mini Nutritional Assessment; MNA-SF, MNA Short Form; ONS, oral nutritional supplements; OR, odds ratio; QoL, quality of life; RCT, randomized controlled trial; RR, risk ratio; SPPB, Short Physical Performance Battery.

## 4 Nursing roles in bridging hospital and home

Nursing participation is documented, but independent nurse-led effectiveness is not established. Three examples in the 13-trial comparison illustrate different roles. In Pedersen, a clinical nurse specialist prepared discharge plans for all groups, while dietitians delivered the experimental follow-up (13, 14). Beck's liaison team included nursing alongside therapy staff; the randomized contrast added a dietitian (25). Terp's trial (*n* = 144) combined a dietitian-prepared plan with community nursing/assistant follow-up at weeks 1, 4, and 8; weight and self-rated health improved, but function, readmission, and mortality did not (4, 29). The personnel description is corroborated by the scoping-review extraction (4); the primary report was available at abstract level. None of these contrasts isolates a nursing contribution.

Across the 13 selected trial families, three provide the clinical-care examples above, and one additional trial, HOMEFOOD, reports nurse involvement in screening/recruitment while a clinical nutritionist delivered the intervention (23). In the remaining nine trial families, the retrieved material did not establish a distinct nursing-care contrast. No identified randomized contrast isolated an independent nursing effect. This classification is limited by purposive selection and incomplete full-text access; unreported nursing involvement cannot be treated as absence. Supplementary material 2, Section S2.3, summarizes these distinctions.

Adjacent evidence also informs role design. In a US home-health quality-improvement program (*n* = 1,546; 67.9% referred from hospitals), nurses or physiotherapists screened patients and implemented a dietitian-developed nutrition protocol (30). Overall hospitalization was lower than in comparison cohorts, but the hospital-origin subgroup showed no significant reduction. Mixed referral routes, non-randomized comparisons, and combined professional delivery prevent nurse-specific or medical-discharge effectiveness claims.

Our proposed nursing functions are readiness assessment, checking portable information and referral status, monitoring intake and barriers, and activating agreed escalation. Patient/carer interviews and cross-setting qualitative evidence support attention to communication and support needs, not causal claims for nursing ownership (10, 31). Teach-back, preferred contact modes, caregiver willingness, and alternatives for patients living alone should inform implementation.

Responsibilities must follow local authorization and competence. Dietitians provide specialist nutrition assessment and individualized treatment; appropriately authorized medical professionals address disease, swallowing or medication-related concerns. Nurses reinforce agreed plans and detect deterioration within their scope; community services confirm capacity and deliver agreed support. Patients and caregivers participate in goals and decisions without assuming unsupported clinical responsibility. Supplementary Table S3 specifies accountable functions, boundaries, and example measures.

Reviews of nursing and non-dietetic nutrition interventions report heterogeneous, low-certainty evidence across settings (32, 33). General nurse-coordinated transitional-care research and integrated-care models provide indirect support for coordination, not nutrition-specific efficacy (34, 35). These bodies of evidence justify testing professional contributions rather than prescribing a universal professional hierarchy.

## 5 Discussion

### 5.1 Why continuity breaks down in practice

Better intake is more consistently observed than broader clinical benefit, but delivery limitations do not fully explain neutral trials. OptiNAM (*n* = 130) reported post-discharge component compliance of 0%−61% and 16-week loss to follow-up of 29% vs. 19%; early intake gains did not translate into superior quality of life (36). Intensified dietetic care (*n* = 76; eligibility ≥55 years, median age 73) showed no between-group benefit and 30% dropout (37). Low adherence is a possible explanation, not proof that full delivery would have worked. Insufficient potency, small samples, competing illness, comparator care, and outcome choice remain alternatives.

The updated CFIR offers a vocabulary for these determinants (7). Innovation Complexity concerns coordinating several tasks; Outer Setting Partnerships & Connections and Financing concern community linkage and funded access; Inner Setting Work Infrastructure, Communications, and Available Resources concern staffing and electronic records; Individuals Capability and Opportunity concern ability to undertake care; and Implementation Process Engaging and Reflecting & Evaluating concern participation and feedback. For example, dietitian interviews describe coordination and resource constraints (38), while electronic-record research describes fragmentation and documentation burden (39). These are our illustrative interpretations of reported findings, not CFIR codes assigned by the original studies or a formal framework analysis.

Home visits permit contextual assessment but require travel and staffing; telephone care may reduce those demands while excluding people with sensory, cognitive, or digital barriers. Plans that depend on free food or an available caregiver may not transfer to under-resourced services. A rural rehabilitation-to-home pilot (15 intervention participants and 15 historical controls) found acceptable family-centred telehealth but an inconclusive primary nutrition-score difference (95% CI −2.07 to 9.01) (40). This adjacent-setting evidence supports feasibility questions, not generalization to acute medical discharge.

Evaluation must separate constructs. Reach is the proportion of eligible patients receiving the intervention; referral completion is one service-process measure. Fidelity is delivery of specified components, whereas adherence concerns patients' use of the agreed plan. Planned, delivered, and received dose describe intended contacts or therapy, actual provision, and actual exposure. Feasibility means practical deliverability; acceptability means perceived agreeableness, not simply workability. Adoption concerns provider/service uptake and sustainability continued routine delivery (8). Supplementary Table S4 gives denominators, timing, and examples; equity is examined through differences in access, burden, and outcomes, not assumed from overall averages.

### 5.2 A practical framework for continuity of nutrition care

We propose six linked service functions, not a guideline, treatment prescription, or validated minimum package. Supplementary Table S2 maps each function to empirical findings, pathway recommendations, and our operational proposal:

Confirm risk, goals, and readiness. Distinguish screening from diagnosis; agree feasible goals and check patient preferences, food access, and caregiver capacity (3, 9, 10).

Create a portable handover. Transfer the authorized plan, barriers, risk signals, referrals, and contact details in usable language (2, 3, 9).

Confirm ownership and access. Obtain acknowledgment from the receiving service and an alternative after rejection or unavailable capacity (3, 31).

Provide early follow-up. Agree an individualized contact interval and accessible mode; record missed contact and its reason. Trials support studying follow-up, not a universal schedule (13, 14, 20, 29).

Escalate unmet needs or deterioration. Specify locally approved triggers, responsible professionals, and response times; do not substitute an algorithm for clinical judgment (9, 10, 31).

Audit implementation and feed back. Review transfer, receipt, contact, escalation closure, patient experience, and inequitable access (3, 8, 28).

The six-step combination, ordering, acknowledgment rules, and escalation-closure measures are author-derived proposals. No cited study tests this complete loop. Figure 1 depicts proposed workflow and feedback, not causal relationships or estimated effects. Adaptation should preserve traceable responsibilities while accommodating dietetic capacity, community services, household resources, and patient choices.

**Figure 1:**
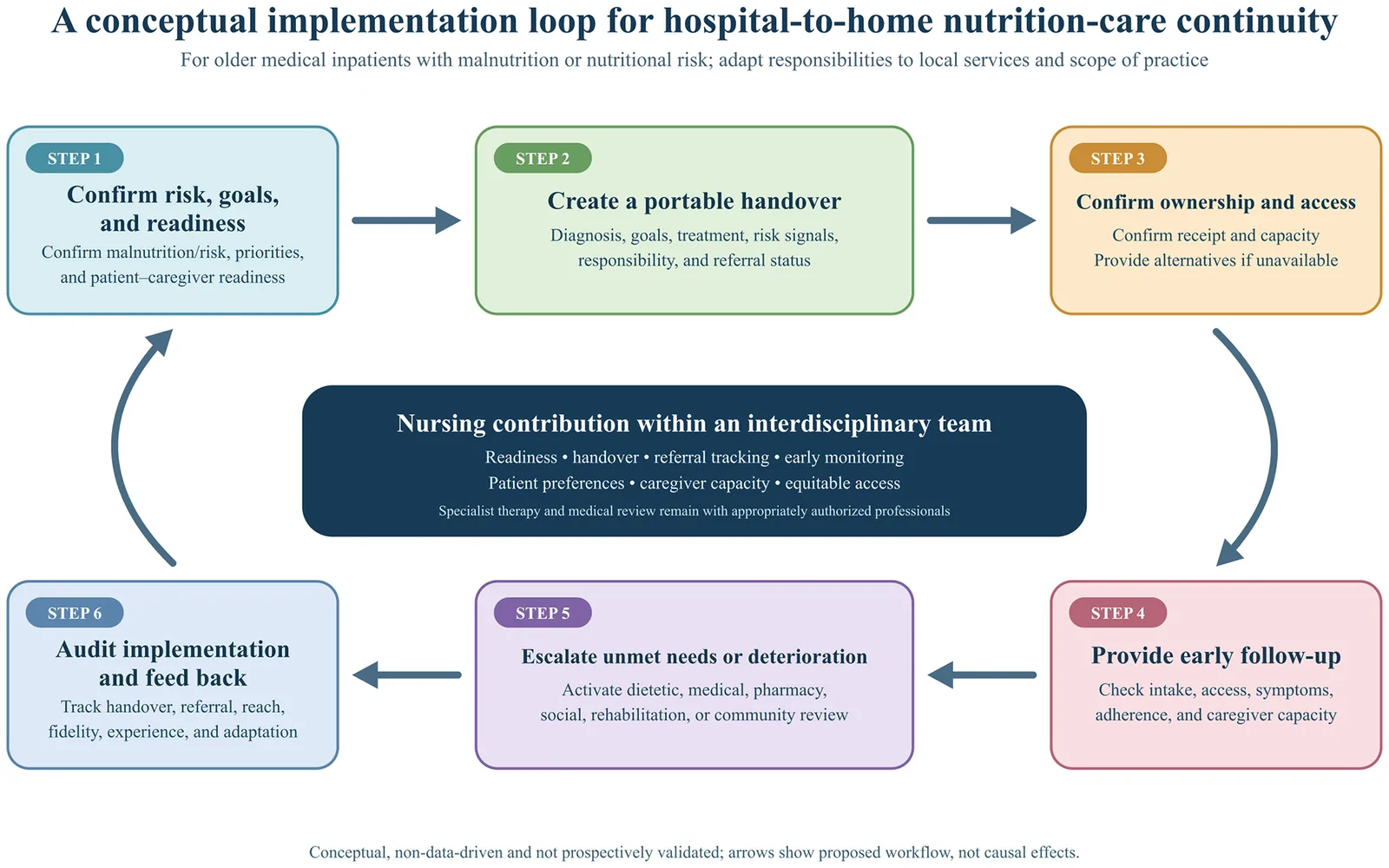
A conceptual implementation loop for hospital-to-home nutrition-care continuity. The six-step loop is a literature-informed, conceptual, non-data-driven framework that has not been prospectively validated. Arrows indicate proposed workflow and feedback, not demonstrated causal effects. Readiness and patient/carer preferences inform all steps. Responsibilities are interdisciplinary and subject to local authorization. Supplementary Tables S2–S4 provide evidence-to-component mapping, role boundaries, and proposed measures.

### 5.3 Next steps for research and service development

Prospective work should co-design roles with patients, caregivers, and providers, then assess feasibility, acceptability, reach, and fidelity before effectiveness claims. Hybrid designs can examine delivery and clinical outcomes together (12). Report staff time, costs, unsuccessful referrals, reasons for declining or discontinuing care, and caregiver burden. Stratify access by living arrangement, functional and cognitive needs, affordability, language/literacy, rurality, and contact-mode access. Test whether adaptations restore access without shifting unsustainable work onto families.

Limitations include purposive rather than exhaustive selection, overlapping databases, absence of subscription and Chinese-database searches, reliance on English-accessible reports, non-duplicated AI-assisted selection/extraction, and incomplete primary-full-text access. Narrative critical appraisal does not replace formal risk-of-bias assessment. Mixed ages, medical/surgical cohorts, adjacent settings, and variable outcome definitions limit directness. Author interpretation may favor recognizable workflow solutions; unmeasured determinants and contrary evidence may remain. The proposed role allocation and loop require prospective evaluation.

## 6 Conclusion

Published evidence documents incomplete transfer and uptake of nutrition care after discharge, while interventions show heterogeneous benefits. Nursing can participate in interdisciplinary planning, coordination, and monitoring, but its independent effect remains uncertain. This review's contribution is a literature-informed conceptual loop linking readiness, handover, acknowledged responsibility, follow-up, escalation, and feedback, with explicit evidence boundaries and proposed measures. Neither the complete loop nor its nursing components should be regarded as validated; prospective evaluation must examine equitable delivery as well as nutritional and clinical outcomes.
