# Supplementary material for: Implementing continuity of nutrition care from hospital to home for older medical inpatients with malnutrition or nutritional risk: nursing contributions, barriers, and practice gaps
Source: Front Nutr. 2026 Oct 22;13:1980311. doi: 10.3389/fnut.2026.1980311 (PMC13736117; doi:10.3389/fnut.2026.1980311)
Supplement: Supplementary file 1 [file Table_1.docx]

Supplementary Material 1

Search strategy and selection record

# S1.1 Review design and scope

This is a focused narrative Mini Review, not a systematic review. The target is older medical inpatients with malnutrition or nutritional risk discharged to a private home, including those receiving community services. Institutional rehabilitation and residential care are not the target destination. Mixed medical/surgical geriatric cohorts, broader age thresholds, and rehabilitation-to-home studies are identified as indirect evidence; their outcomes are not attributed specifically to older medical patients.

This supplement documents a focused narrative selection, not an exhaustive systematic review. Original empirical studies were prioritized when they informed documented continuity failures, comparison of transition components, or professional and organizational delivery. Reviews, consensus pathways, implementation-method papers and a study protocol provide explicitly contextual support. Narrow disease-specific nutrition regimens, inpatient-only treatment without a transition function, long-term institutional care and unrelated nutritional epidemiology were not the focus. Mixed medical/surgical geriatric populations and broader age criteria were retained only with their indirectness identified. One rehabilitation-to-home pilot informs a bounded discussion of family-centred rural delivery, not medical-inpatient effectiveness.

# S1.2 Search dates and retrieval: Supplementary Table S1

| **Search** | **Retrieved records** | **Role** |
| --- | --- | --- |
| Q1_PubMed_broad | 493 | Broad biomedical retrieval |
| Q2_PubMed_focused | 123 | Focused continuity terminology |
| Q3_PubMed_nursing | 167 | Expanded nursing branch |
| Q4_PubMed_dietetic_sensitivity | 167 | Dietetic counselling food and ONS sensitivity branch |
| PubMed unique records | 776 | 950 records across branches; 174 repeated occurrences removed by PMID |
| Europe PMC | 7 | 6 overlapping PubMed IDs; 1 additional record by database ID |

Queries were executed for the revision with a publication-date upper bound of 14 September 2026. Complete PubMed result sets and article metadata/abstracts were exported, not only a relevance-ranked first page. Search counts are database outputs, not counts of eligible studies. Europe PMC substantially overlaps PubMed and is not treated as an independent comprehensive biomedical database.

# S1.3 Exact queries

PubMed field tags: [tiab] = title/abstract; [Mesh] = Medical Subject Headings; [Date - Publication] = publication date. An asterisk is a literal truncation wildcard. No language or publication-type filter was added to the revision queries; interpretation was limited by English-accessible source material.

## Q1_PubMed_broad

(( ("Nutrition Therapy"[Mesh] OR "Nutritional Support"[Mesh] OR "nutrition care"[tiab] OR "nutritional care"[tiab] OR "nutrition support"[tiab] OR "nutrition intervention*"[tiab] OR "nutrition plan*"[tiab] OR "nutrition pathway*"[tiab]) AND ("Patient Discharge"[Mesh] OR "Continuity of Patient Care"[Mesh] OR "discharge planning"[tiab] OR "hospital to home"[tiab] OR "hospital-to-home"[tiab] OR "hospital to community"[tiab] OR "hospital-to-community"[tiab] OR postdischarg*[tiab] OR "care transition*"[tiab] OR "transitional care"[tiab] OR "care continuity"[tiab] OR referral*[tiab] OR follow-up[tiab]) AND ("Aged"[Mesh] OR "Aged, 80 and over"[Mesh] OR "older adult*"[tiab] OR "older patient*"[tiab] OR elder*[tiab] OR geriatric*[tiab]) AND ("Malnutrition"[Mesh] OR malnutrition[tiab] OR malnourish*[tiab] OR undernutrition[tiab] OR "nutrition risk"[tiab] OR "nutritional risk"[tiab]) )) AND ("1900/01/01"[Date - Publication] : "2026/09/14"[Date - Publication])

## Q2_PubMed_focused

(( ("nutrition care"[tiab] OR "nutritional care"[tiab] OR "nutrition support"[tiab] OR "nutrition intervention*"[tiab] OR "nutrition plan*"[tiab] OR "nutrition pathway*"[tiab]) AND ("hospital to home"[tiab] OR "hospital-to-home"[tiab] OR "hospital to community"[tiab] OR "hospital-to-community"[tiab] OR ((discharg*[tiab] OR "care transition*"[tiab] OR "transitional care"[tiab]) AND (hospital*[tiab] OR inpatient*[tiab] OR "acute care"[tiab]))) AND ("older adult*"[tiab] OR "older patient*"[tiab] OR elder*[tiab] OR geriatric*[tiab] OR "Aged"[Mesh]) AND (malnutrition[tiab] OR malnourish*[tiab] OR undernutrition[tiab] OR "nutrition risk"[tiab] OR "nutritional risk"[tiab] OR "Malnutrition"[Mesh]) )) AND ("1900/01/01"[Date - Publication] : "2026/09/14"[Date - Publication])

## Q3_PubMed_nursing

((("Nurses"[Mesh] OR "Nursing Care"[Mesh] OR nurs*[tiab]) AND (malnutri*[tiab] OR malnourish*[tiab] OR undernutri*[tiab] OR "nutritional risk"[tiab] OR "Malnutrition"[Mesh]) AND ("Patient Discharge"[Mesh] OR "Transitional Care"[Mesh] OR discharg*[tiab] OR transition*[tiab] OR "hospital to home"[tiab]) AND ("Aged"[Mesh] OR older[tiab] OR elder*[tiab] OR geriatric*[tiab]))) AND ("1900/01/01"[Date - Publication] : "2026/09/14"[Date - Publication])

## Q4_PubMed_dietetic_sensitivity

(((dietiti*[tiab] OR dietetic*[tiab] OR "dietary counselling"[tiab] OR "dietary counseling"[tiab] OR "oral nutritional supplement*"[tiab] OR "food delivery"[tiab] OR "meal delivery"[tiab]) AND (discharg*[tiab] OR "hospital to home"[tiab] OR "hospital-to-home"[tiab] OR "transitional care"[tiab]) AND ("Aged"[Mesh] OR older[tiab] OR elder*[tiab] OR geriatric*[tiab]) AND ("Malnutrition"[Mesh] OR malnutri*[tiab] OR malnourish*[tiab] OR undernutri*[tiab] OR "nutritional risk"[tiab]))) AND ("1900/01/01"[Date - Publication] : "2026/09/14"[Date - Publication])

## Europe PMC focused query

(TITLE_ABS:("nutrition care" OR "nutritional care" OR "nutrition support" OR "nutrition plan" OR "nutrition pathway") AND TITLE_ABS:("hospital to home" OR "hospital-to-home" OR "hospital to community" OR "hospital-to-community" OR postdischarge OR "post-discharge" OR "care transition" OR "transitional care") AND TITLE_ABS:(malnutrition OR malnourished OR "nutritional risk") AND TITLE_ABS:("older adult" OR elderly OR geriatric)) AND FIRST_PDATE:[1900-01-01 TO 2026-09-14]

Europe PMC uses TITLE_ABS and FIRST_PDATE. The result set was retrieved in full. No subscription-database search is implied by locating a publisher page or a PubMed-indexed paper.

# S1.4 Targeted selection and missed study checks

All 776 PubMed titles were inspected. Abstracts were examined for plausible relevance and informative contrasts, followed by accessible full-text checks. This was a purposive, non-duplicated, AI-assisted process; it was not an independently adjudicated inclusion/exclusion workflow. Priorities were directness to acute medical/geriatric discharge, clear comparator and outcome reporting, documentation of delivery, and information on null results or practical barriers. Bibliographic records were verified by PMID/DOI. The final 41 references are cited publications, including contextual sources, companion reports and a published corrigendum, not a count of eligible studies or independent cohorts.

Reference lists and evidence tables of relevant reviews, particularly Ingstad and Sarier, were inspected for missed studies and linked reports. Targeted title/DOI checks were used to examine potential omissions, personnel descriptions and companion publications. The expanded nursing and dietetic branches provided additional terminology coverage. This was backward reference/evidence-table checking and targeted verification, not a formal forward-citation search or an exhaustive citation network.

During revision, eight publications were added to the original submission's reference list: Beck 2013 and 2015, McMurdo 2009, Söderström 2020, Terp 2018, Riley 2020, a primary HOMEFOOD nutritional/functional report, and Marshall 2026. Their roles and limitations are shown in Table 1 and Supplementary Material 2. These additions are selected contrasts, not all potentially relevant publications in the 776-record export. Companion reports are grouped: Pedersen has two papers and HOMEFOOD three. No pooled participant count is calculated.

During proof checking, the published corrigendum to the HOMEFOOD service-outcome report (41; DOI: 10.1016/j.jnha.2024.100456; PMID: 39787989) was identified and its full text checked. It was added to the 40 references retained at revision, bringing the final citation count to 41. The corrigendum corrects outcomes from the existing HOMEFOOD trial; it is not an additional independent study. The comparison therefore remains 13 selected trial families. This targeted proof-stage verification does not change the search cutoff of 14 September 2026 or the retrieval counts in Supplementary Table S1. The corrected findings and source-access record are documented in Supplementary Material 2, Section S2.5.

# S1.5 Limitations and historical search record

The original search through 21 August 2026 used relevance-prioritized retrieval and targeted missed-study checking; it did not retain a complete record-level selection history. No historical screening or exclusion counts have been reconstructed. The fully exported revision search supersedes that record for reproducibility. Some truncation asterisks were lost when the earlier search supplement was formatted as Word text; the executable strings above preserve them literally. This formatting issue alone does not establish which query was executed historically.

PubMed was used for biomedical coverage and Europe PMC for complementary retrieval/full-text access. CINAHL would add discipline-specific nursing coverage; Embase and Chinese databases could identify further studies. They were not searched, and the two accessible sources are not claimed to replace them. English-accessible interpretation, selective full-text availability, purposive prioritization, lack of duplicate selection/extraction and absence of formal risk-of-bias assessment may introduce selection and interpretive bias. These limitations are retained in the main manuscript.
