# Supplementary material for: Implementing continuity of nutrition care from hospital to home for older medical inpatients with malnutrition or nutritional risk: nursing contributions, barriers, and practice gaps
Source: Front Nutr. 2026 Oct 22;13:1980311. doi: 10.3389/fnut.2026.1980311 (PMC13736117; doi:10.3389/fnut.2026.1980311)
Supplement: Supplementary file 2 [file Table_2.docx]

Supplementary Material 2

Evidence appraisal and implementation mappings

Reference numbers correspond to the revised main manuscript. These materials support a focused narrative synthesis; they are not a systematic evidence inventory, formal risk-of-bias assessment, validated clinical pathway, or new empirical dataset.

# S2.1 Supplementary observational and adjacent setting evidence

## Young 2015 (1)

Australia; prospective medical-discharge cohort; n=42; ≥65, nutritional risk; routine care; nutrition/function 6 weeks and service outcomes 12 weeks.

Six received dietetic review and eight practical support. Small cohort and lack of comparator limit generalization; do not treat its follow-up rates as population estimates.

## Brooks 2019 (2)

US; retrospective chart review; n=76 malnourished adults; mixed ages; discharge instructions.

Five had ONS instructions. Documentation may under-record actual advice; indirect age coverage. Shows a documentation problem, not effect of a corrected handover.

## Gomes 2024 (3)

Australia; retrospective medical/surgical audit; 345 admissions in 306 patients ≥65; documented malnutrition; follow-up to 6 months.

35/345 admissions had nutrition recommendations in discharge summaries; 79/345 had recorded dietetic follow-up. Admission-level denominators must not be labelled unique patients. Care outside the record and mortality complicate interpretation.

## Hestevik 2020 (10)

Norway; qualitative interviews; 15 older patients and nine caregivers; home nursing after geriatric hospitalization.

Food preferences, difficulties, information and involvement were salient. Rich patient perspective; one urban care context and purposive sample cannot estimate prevalence or effectiveness.

## Young 2018 (18)

Australia; dietitian-led pre–post HHOME program at two hospitals; ≥65 medical patients; four-week calls; outcomes 6 and 12 weeks.

Nutritional change was not significantly different; gait-speed change favored HHOME. Non-randomized temporal comparison and small samples prevent causal attribution. The pre-HHOME cohort may overlap the earlier service research; no sample sizes are pooled.

## Hamirudin 2017 (19)

Australia; 68 Department of Veterans’ Affairs clients ≥65; single-dietitian home intervention; baseline within 2 weeks and follow-up at 3 months.

Within-group weight and MNA improved. No parallel comparator; not all participants were underweight. Feasibility in this selected entitlement/service context is not proof of effectiveness or general reach.

## Sanchez 2026 (28)

France; retrospective publicly funded program; n=351; mean age 87.3; planned 4–5 visits over 3 months.

197 had ≥2 visits, 189 weight-change data, 149 all visits. Caregiver number associated with completion. Attrition/completer selection and natural recovery limit interpretation of weight gain.

## Verwijs 2020 (31)

Netherlands; cross-setting qualitative study with older adults, carers and 41 professionals; nutritional risk not required for all participants.

Communication, knowledge, food provision and accountability themes. Includes rehabilitation/community perspectives; indirect to malnourished medical-discharge patients. Themes are not frequencies or causal determinants.

## Gomes 2025 (38,39)

Australia; two reports on dietitian perspectives, discharge workflows and integrated electronic records.

Reported coordination/resource constraints and fragmented documentation support organizational hypotheses. Related investigators/settings may overlap; reports are not treated as independent replications. They used their own analytic approaches, not our CFIR interpretation.

## Riley 2020 (30)

US; home-health pre–post QIP; n=1546 versus historic n=7413 and concurrent n=5235; mean age 76.8; 67.9% hospital, remainder outpatient/SNF referrals; 90 days.

Nurses/physiotherapists screened and used a dietitian-designed protocol. Overall hospitalization associations favored QIP; hospital-origin subgroup reductions were not significant. Non-randomization, mixed ages/routes and commercial support constrain causal and economic inference.

## Marshall 2026 (40)

Australia; rural rehabilitation-to-home pilot; 15 patient/carer dyads versus 15 historical controls; three months after discharge.

Primary PG-SGA difference 3.46 (95% CI −2.07 to 9.01), inconclusive; categorical nutrition outcome favored intervention and acceptability was reported. Small historical comparison and different discharge setting; supports cautious co-design/feasibility discussion, not acute-medical effectiveness.

# S2.2 Evidence-to-component mapping

**Supplementary Table S2 Evidence to component mapping**

| **Proposed function** | **Empirical or contextual basis** | **Our interpretation and operational proposal** | **Evidence boundary** |
| --- | --- | --- | --- |
| 1 Confirm risk, goals, and readiness | Audits (2,3); patient/carer interviews (10); consensus pathway (9). | Agree goals and check food access, functional capacity, preferences and caregiver willingness before transfer. | Needs are observed; the combined readiness checklist and its thresholds are not validated. |
| 2 Create a portable handover | Documented gaps (2,3); pathway recommendations (9,11). | Transfer diagnosis/risk, authorized plan, barriers, contacts and outstanding actions in accessible language. | Documentation audits support the problem; this minimum dataset is an author-derived proposal. |
| 3 Confirm ownership and access | Referral uptake audit (3); communication and accountability themes (31); protocol (12). | Record service acknowledgement, responsible contact and alternatives after rejection/non-response. | Acknowledgement rules and causal benefits have not been tested here; the protocol provides no outcome evidence. |
| 4 Provide early follow-up | Comparative trials (13,14,20,24,25,29); routine completion data (28). | Agree time and mode; document offered, delivered and received contact; adapt to access barriers. | Follow-up components have trial support with mixed results; no universal schedule or isolated mode effect is established. |
| 5 Escalate unmet needs or deterioration | Patient/carer difficulties (10), coordination themes (31), pathway recommendations (9,11). | Agree local deterioration triggers, dietetic/medical response and documented closure; include food-access and caregiver crises. | Operational escalation rules are proposed, not a tested triage algorithm or treatment protocol. |
| 6 Audit implementation and feed back | Observed documentation and completion gaps (3,28); implementation-outcome concepts (8). | Review process denominators, patient experience, reach and inequities; feed findings to the responsible teams. | Outcome concepts are established; benefit of this audit-feedback loop has not been demonstrated. |

# S2.3 Nursing participation and interdisciplinary roles

Within the 13 selected trial families in Table 1, three clinical-care examples document nursing participation: Pedersen (13,14), Beck 2015 (25), and Terp (4,29). One additional trial, HOMEFOOD (23), reports attending nurses collaborating in screening/recruitment while a clinical nutritionist delivered the intervention. In the remaining nine trial families, the retrieved material did not establish a distinct nursing-care contrast. No identified randomized contrast isolated an independent nursing effect. These categories describe the available reports, not a formal coding analysis or a field-wide inventory. Unreported nursing involvement cannot be treated as absence. Beck 2015 remains supported by an abstract and partial team excerpt; Terp personnel details remain secondary-corroborated. The source register in Section S2.5 retains these access limits.

Outside this 13-trial denominator, Riley (30) provides one adjacent, non-randomized nurse/physiotherapist-delivered home-health QIP with mixed referral routes. Its hospital-origin subgroup did not show a significant hospitalization reduction. Four contextual reviews (32–35) provide indirect, cross-setting support, not four additional independent nutrition-transition trials. Qualitative studies (10,31) inform communication and support needs. The role assignments and measures in Supplementary Table S3 remain proposals for prospective evaluation.

**Supplementary Table S3 Proposed interdisciplinary role matrix**

| **Role** | **Proposed contribution** | **Boundary and handover** | **Example evaluation** |
| --- | --- | --- | --- |
| Nurses | Assess practical readiness; reinforce agreed plan; verify handover/referral state; observe intake, symptoms and missed services. | Act within local competence and authorization. Refer specialist assessment/therapy decisions to appropriately qualified clinicians; do not inherit unresourced dietetic work. | Handover completeness; unresolved-referral closure; escalation response; staff time. Evidence: (10,13,14,29,30,31); proposed allocation, not proof of nursing efficacy. |
| Dietitians | Specialist assessment; individualized nutrition therapy; adaptation to preferences and affordability; monitoring and education. | Record the authorized plan, expected monitoring and thresholds; coordinate medical review for underlying causes. Local professional rules apply. | Timely specialist contact; appropriate plan revision; patient-understood goals; nutrition outcomes. Evidence: (9,13,14,20,24,25). |
| Physicians and authorized advanced practitioners | Evaluate medical contributors and deterioration; review medication/disease restrictions; authorize treatment within local rules. | Urgent medical evaluation must not be delayed by routine nutrition referral. Escalation authority and coverage must be agreed locally. | Time to clinically indicated review and closure; adverse events. Basis: interdisciplinary pathways (9,11); operational proposal. |
| Primary and community services | Accept or decline referrals explicitly; name a contact; provide agreed home visits, meals, transport/social support or dietetic care. | State eligibility, capacity and funding constraints; notify referrer/patient when service cannot be provided. | Acceptance and first-contact intervals; unmet need; equitable access. Evidence: (3,9,28,31); acknowledgement rule proposed. |
| Patients | Set goals/preferences; decide whether the plan is acceptable; report barriers and concerns using an accessible route. | A person may decline or request changes. Inability to buy food, use technology or manage symptoms is not simply non-adherence. | Goal concordance, acceptability, treatment burden and access. Evidence: (10,31); proposed measures. |
| Caregivers | Participate with consent; support meals, communication and monitoring if willing and able. | Do not assume every patient has a caregiver or transfer untrained clinical duties to family; offer alternatives and assess burden. | Caregiver willingness, burden, support needs and continuity when absent. Evidence: (10,28,40); proposed measures. |

# S2.4 Implementation measurement framework

**Supplementary Table S4 Proposed measurement framework**

Definitions draw on implementation-outcome concepts (8), but the operational examples, denominators and timing specifications are proposed for prospective local evaluation. They are not validated thresholds.

| **Construct or process** | **Operational example for prospective testing** | **Interpretive safeguard** |
| --- | --- | --- |
| Reach | Number of eligible home discharges receiving the defined intervention divided by all eligible home discharges, within a prespecified period; report offered and enrolled separately. | A rate among enrollees is not population reach. Report refusal, death, ineligibility and loss separately. |
| Referral completion | Accepted referrals and completed first contacts divided by referrals made; report both states with dates and unresolved reasons. | Do not substitute referral placement for care received or for overall reach. |
| Fidelity | Required components delivered as specified divided by applicable prescribed components; audit handover, acknowledgement and escalation closure. | Fidelity concerns provider delivery; distinguish permitted adaptation from omission. |
| Patient adherence | Patient use of the agreed food/ONS/monitoring plan over a defined interval; record method and denominator (e.g., doses reported consumed/prescribed). | Do not blame the patient for unavailable products/services; self-report and survivorship can bias estimates. |
| Planned delivered and received dose | Record scheduled contact number/duration; contacts actually delivered; contacts attended/usable exposure and actual product consumption separately. | A completed appointment does not prove nutrition advice was understood or applied. |
| Feasibility | Staff time, available workforce, cost, successful scheduling and practical ability to deliver within existing resources. | Feasible for a trial team is not necessarily feasible in routine service. |
| Acceptability | Patient, caregiver and staff assessments of agreeableness, satisfaction and burden, using a stated method and time point. | Acceptability is not a synonym for feasibility or clinical benefit. Include people who decline/discontinue. |
| Adoption | Eligible services or clinicians initiating the defined workflow divided by those invited/eligible; specify unit and period. | Do not confuse provider uptake with patient adherence. |
| Sustainability | Continued delivery and fidelity at prespecified later intervals after initial implementation support is reduced. | Longer patient follow-up alone does not demonstrate sustained routine implementation. |
| Equity across outcomes | Compare reach, referral delays, discontinuation, burden and clinical outcomes by relevant access needs and resources. | Equity is cross-cutting; subgroup differences describe gaps but do not by themselves establish causes. |

# S2.5 Source verification and evidence boundaries

The original 40 cited publications were checked against PubMed identifiers and bibliographic/abstract records. The published HOMEFOOD corrigendum (41) was added and its bibliographic record and full text checked during proof review, bringing the final citation count to 41. Access is recorded below. “Primary full text available” denotes a retrieved source used for targeted checks, not a formal appraisal of every reported outcome. For abstract-limited reports, table entries and claims are restricted to accessible information; personnel details from review extraction are separately acknowledged. These checks were AI-assisted and not independently duplicated.

HOMEFOOD proof-stage update (22,41): The corrigendum corrects a hospital-record linkage error affecting readmission counts and length of stay. Corrected analyses showed significantly fewer readmissions and shorter length of stay at 1 month under hospital-care definitions A-C, but no significant differences at 6, 12 or 18 months. Including day-hospital post-acute rehabilitation (definition D) also removed the 1-month differences. These findings do not establish a sustained readmission benefit and replace the original service-outcome interpretation. The corrigendum concerns the same trial reported in references 21-23; it does not add a trial or alter the 13-trial denominator or the nursing-role classification in Section S2.3.

| **Ref.** | **Study or source** | **Access and use boundary** |
| --- | --- | --- |
| 1 | Young AM \| 2015 PMID 27032047 | PubMed bibliographic record/abstract; primary full text not available for this synthesis. |
| 2 | Brooks M \| 2019 PMID 31037779 | PubMed bibliographic record/abstract; primary full text not available for this synthesis. |
| 3 | Gomes K \| 2024 PMID 39203932 | Primary full text available for targeted verification. |
| 4 | Ingstad K \| 2020 PMID 33148761 | Primary full text available for targeted verification. Contextual source; not counted as a new outcome study. |
| 5 | Sarier C \| 2025 PMID 41097258 | Primary full text available for targeted verification. Contextual source; not counted as a new outcome study. |
| 6 | Munk T \| 2016 PMID 25786644 | PubMed bibliographic record/abstract; primary full text not available for this synthesis. Contextual source; not counted as a new outcome study. |
| 7 | Damschroder LJ \| 2022 PMID 36309746 | Primary full text available for targeted verification. Contextual source; not counted as a new outcome study. |
| 8 | Proctor E \| 2011 PMID 20957426 | Primary full text available for targeted verification. Contextual source; not counted as a new outcome study. |
| 9 | Keller H \| 2022 PMID 33417240 | PubMed bibliographic record/abstract; primary full text not available for this synthesis. Contextual source; not counted as a new outcome study. |
| 10 | Hestevik CH \| 2020 PMID 32206325 | Primary full text available for targeted verification. |
| 11 | Ng DHL \| 2023 PMID 37960145 | Primary full text available for targeted verification. Contextual source; not counted as a new outcome study. |
| 12 | Murofushi K \| 2026 PMID 41877291 | Primary full text available for targeted verification. Contextual source; not counted as a new outcome study. |
| 13 | Pedersen JL \| 2016 PMID 27709234 | Primary full text available for targeted verification. |
| 14 | Lindegaard Pedersen J \| 2017 PMID 27999853 | Primary full text available for targeted verification. |
| 15 | Deutz NE \| 2016 PMID 26797412 | PubMed bibliographic record/abstract; primary full text not available for this synthesis. |
| 16 | Feldblum I \| 2011 PMID 21087222 | PubMed bibliographic record/abstract; primary full text not available for this synthesis. |
| 17 | Sharma Y \| 2017 PMID 28472530 | PubMed bibliographic record/abstract; primary full text not available for this synthesis. |
| 18 | Young AM \| 2018 PMID 29392865 | PubMed bibliographic record/abstract; primary full text not available for this synthesis. |
| 19 | Hamirudin AH \| 2017 PMID 28731606 | PubMed bibliographic record/abstract; primary full text not available for this synthesis. |
| 20 | Munk T \| 2021 PMID 34656032 | PubMed bibliographic record/abstract; primary full text not available for this synthesis. |
| 21 | Blondal BS \| 2022 PMID 35331537 | PubMed bibliographic record/abstract; primary full text not available for this synthesis. |
| 22 | Blondal BS \| 2023 PMID 37702336 | Primary full text available for targeted verification. Service outcomes are interpreted using the published corrigendum (41), checked during proof review; see the HOMEFOOD update above. |
| 23 | Blondal BS \| 2023 PMID 36028775 | Primary full text available for targeted verification. |
| 24 | Beck AM \| 2013 PMID 23258932 | PubMed bibliographic record/abstract; primary full text not available for this synthesis. |
| 25 | Beck A \| 2015 PMID 25552522 | PubMed bibliographic record/abstract; primary full text not available for this synthesis. Liaison personnel corroborated in an author-posted full-text excerpt; not a complete primary-text appraisal. |
| 26 | McMurdo ME \| 2009 PMID 19925613 | PubMed bibliographic record/abstract; primary full text not available for this synthesis. |
| 27 | Söderström L \| 2020 PMID 32362168 | Primary full text available for targeted verification. |
| 28 | Sanchez M \| 2026 PMID 41851837 | Primary full text available for targeted verification. |
| 29 | Terp R \| 2018 PMID 29606025 | PubMed bibliographic record/abstract; primary full text not available for this synthesis. Personnel description corroborated by Ingstad’s scoping-review table, not the primary full text. |
| 30 | Riley K \| 2020 PMID 31231830 | Primary full text available for targeted verification. |
| 31 | Verwijs MH \| 2020 PMID 32525265 | Primary full text available for targeted verification. |
| 32 | van den Berg GH \| 2021 PMID 33647842 | PubMed bibliographic record/abstract; primary full text not available for this synthesis. Contextual source; not counted as a new outcome study. |
| 33 | Dabbous M \| 2021 PMID 34224986 | PubMed bibliographic record/abstract; primary full text not available for this synthesis. Contextual source; not counted as a new outcome study. |
| 34 | Parker KJ \| 2024 PMID 38951122 | PubMed bibliographic record/abstract; primary full text not available for this synthesis. Contextual source; not counted as a new outcome study. |
| 35 | Leithaus M \| 2022 PMID 35855092 | Primary full text available for targeted verification. Contextual source; not counted as a new outcome study. |
| 36 | Andersen AL \| 2024 PMID 38777473 | PubMed bibliographic record/abstract; primary full text not available for this synthesis. |
| 37 | van Dronkelaar C \| 2025 PMID 39667560 | PubMed bibliographic record/abstract; primary full text not available for this synthesis. |
| 38 | Gomes K \| 2025 PMID 41102938 | PubMed bibliographic record/abstract; primary full text not available for this synthesis. |
| 39 | Gomes K \| 2025 PMID 40941579 | Primary full text available for targeted verification. |
| 40 | Marshall S \| 2026 PMID 42294533 | Primary full text available for targeted verification. |
| 41 | Blondal BS \| 2025 HOMEFOOD corrigendum PMID 39787989 DOI 10.1016/j.jnha.2024.100456 | Bibliographic record and primary corrigendum full text checked during proof review (PMCID PMC12180053). Corrects the service outcomes of reference 22; not an additional independent study. See the HOMEFOOD update above. |

# S2.6 Scope of critical appraisal

The appraisal addresses design, sample selection, age/setting directness, comparator care, attrition, follow-up, endpoint specificity, precision and attribution of bundled components. No numerical quality score, RoB 2 judgment, GRADE certainty rating or formal CFIR coding has been assigned. An abstract-only report does not justify assertions about unreported allocation concealment, blinding, fidelity or subgroup methods. Narrative statements of potential bias are interpretive limitations, not formal risk-of-bias judgments.
